# Cationic nanomicelles derived from Pluronic F127 as delivery vehicles of Chinese herbal medicine active components of ursolic acid for colorectal cancer treatment†

**DOI:** 10.1039/c8ra01071d

**Published:** 2018-04-27

**Authors:** Zhaokun Yan, Qingtang Wang, Xiaolong Liu, Jun Peng, Qin Li, Ming Wu, Jiumao Lin

**Affiliations:** Academy of Integrative Medicine, Fujian University of Traditional Chinese Medicine Fuzhou 350122 P. R. China jiumaolin@hotmail.com; The United Innovation of Mengchao Hepatobiliary Technology Key Laboratory of Fujian Province, Mengchao Hepatobiliary Hospital of Fujian Medical University Fuzhou 350025 P. R. China wmmj0419@163.com; The Liver Center of Fujian Province, Fujian Medical University Fuzhou 350025 P. R. China; Fujian Key Laboratory of Integrative Medicine on Geriatrics, Fujian University of Traditional Chinese Medicine Fuzhou Fujian 350122 P. R. China

## Abstract

Ursolic acid (UA) has shown great potential in cancer therapy but their efficacy is seriously compromised by poor water-solubility and limited cellular uptake. In this paper, cationic nanomicelles self-assembled from Pluronic F127 with the cationic polymer of C_18_-polyethylenimine (C_18_-PEI) as a functional component are fabricated as delivery vehicles of Chinese herbal medicine active components of ursolic acid (UA) for colorectal cancer treatment. The inhibition effects of this drug loaded cationic nanomicelles (named as FUP) on cell viability and cell colony formation were more significant than the free UA, due to their cationic surface which can increase UA uptake by colorectal cancer cells. Cell cycle analysis showed that this inhibition effect was mediated by a cell cycle arrest at the G1 checkpoint, and the cell death induced by these nanomicelles occurred *via* apoptosis, which was detected by annexin V antibody and propidium iodide staining. Further western blot analysis demonstrated the apoptosis mechanism was associated with the regulation of Fas/FasL and activation of caspase-8 and caspase-3. Therefore, our cationic nanomicelles can potentially be used to enhance the therapeutic effect of UA for colorectal cancer treatment.

## Abstract 2

A cationic nanomicelle derived from Pluronic F127 as a delivery vehicle of ursolic acid for enhanced colorectal cancer treatment.

## Graphical abstract

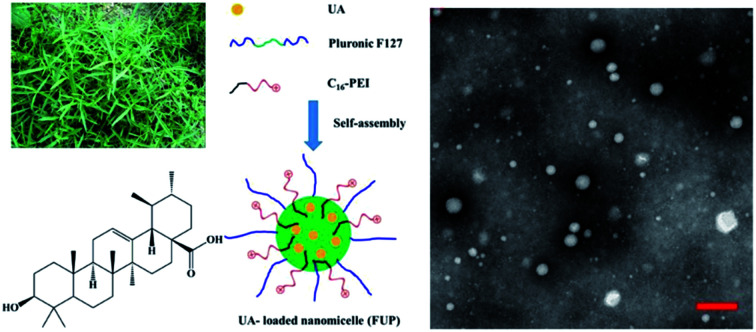

## Introduction

Colorectal cancer (CRC) is the third most frequent type of cancer in males and the second in females, and the fourth most common cause of oncological death.^1,2^ Surgical resection of the tumour-bearing and adjacent segments of the intestine is still the most common treatment for CRC patients. However, about half of patients are diagnosed beyond stage III, whereas invasion of the intestinal wall and metastasis to surrounding tissues have already occurred. To overcome the deficiency of surgical treatment, chemotherapy is often used as an adjuvant therapy to alleviate the symptoms and prolong survival.^3,4^ Unfortunately, most of the current chemotherapeutic agents including 5-fluorouracil, leucovorin, oxaliplatin, irinotecan and capecitabine have serious side-effects, such as anemia, leucopenia, thrombocytopenia and peripheral neuropathy, resulting in limited dosage administration and severely decreased therapeutic efficacy.^5–8^ Compared with the current clinical chemotherapeutic drugs, traditional Chinese medicine recognized as multi-components and multi-target agents can exert their therapeutic function in a more holistic way, thus these naturally-derived agents are promising candidates for anti-cancer treatment.^9,10^

Ursolic acid (UA), a pentacyclic triterpenoid extracted from *Hedyotis diffusa* Willd, has gained extensive interest for its anti-inflammatory, hepatoprotection and antitumor properties in colon cancer cells, endometrial cancer cells, and melanoma cells.^11–13^ Previous studies have reported that UA can regulate the STAT3, ERK, JNK and p38 pathways to reduce the expressions of cyclin D1 and CDK4 and increase the expression of p21, resulting in the growth inhibition of colorectal cancer cell and abduction of the cell apoptosis by increasing the ratio of Bcl-2 to Bax.^14^ However, the clinical application of UA is extremely hindered by some drawbacks. For instance, the limited water solubility of UA leads to the low bioavailability and poor pharmacokinetics *in vivo*, which subsequently restricts its efficacy.

In recent years, it has been considered that polymeric micelles are promising carriers for anticancer drugs.^15,16^ Polymeric micelles are nano-scaled structures formed by amphiphilic block copolymers composed of hydrophilic and hydrophobic chains through self-assembly role in water.^17^ Compared with free drugs, nanocarriers exhibit higher accumulation in solid tumors through the enhanced permeability and retention (EPR) effect.^18^ Among various polymeric micelles, Pluronic F127 is a wide-used biocompatible polymeric drug delivery vehicle, comprising of polyethylene oxide–polypropylene oxide–polyethylene oxide (PEO–PPO–PEO) chains.^17^ In detail, the PPO segment of Pluronic F127 self-assembles into a hydrophobic core for incorporation of lipophilic drugs, while hydrophilic PEO segment of F127 prevents the adsorption and aggregation with other bio-macromolecules.^18^ However, like a double-edged sword, the PEO segment in Pluronic F127 appears to hinder the cellular internalization of nanomicelles and therefore becomes to be an obstacle in the full realization of therapy effects.^19,20^ Compared with nonionic nanovehicles, the positively charged nanoparticles have strong affinity with negatively charged cell membranes which result in high cellular uptake.^21,22^

In the present study, we reported an efficient UA delivery system (FUP) with Pluronic F127 as the drug carrier while cationic polymer of stearoyl chloride grafted polyethylenimine copolymer (C_18_-PEI) as an functional adjuvant to increase the cellular uptake which can bring out the enhanced therapeutic effect. The physico-chemical properties of the obtained nanoparticles were characterized by various techniques such as transmission electron microscopy (TEM), dynamic light scattering (DLS) measurement, fourier transform infrared (FT-IR) spectrum. The drug loading and release behaviour were determined by HPLC. Furthermore, MTT assay, cell cycle analysis, colony formation assay were used to demonstrate the enhanced antitumor efficacy of this cationic drug delivery system, compared with free FUP. Meanwhile, annexin V-FITC/propidium iodide staining and western blot assay were also employed to elucidate that the cell death induced by our carriers was occurred through apoptotic pathways.

## Experimental section

### Materials

UA was purchased from Aladdin (Shanghai, China). Pluronic F127 was purchased from Sigma-Aldrich (St. Louis, MO, USA). C_18_-PEI was synthesized from our previous methods.^22^ 3-[4,5-Dimethylthiazol-2-yl]-2,5-diphenyl-tetrazolium bromide (MTT) were obtained from Solarbio Science & Technology (Beijing, China). Annexin V-fluoroisothio cyanate (FITC)/propidium iodide (PI) apoptosis detection kit, Cell Cycle and Apoptosis Analysis Kit and RIPA lysis buffer and blocking buffer were obtained from Beyotime (Shanghai, China). Rabbit polyclonal antibodies against caspase-3 and caspase-8 were purchased from Cell Signaling Technology (Beverly, MA, USA). Rabbit polyclonal antibodies against Fas and FasL were procured from Biosynthesis Biotechnology (Beijing, China). Rabbit polyclonal antibodies against β-actin were supplied by Proteintech (Wuhan, China). HRP-conjugated goat anti-rabbit was from Earthox (Millbrae, CA, USA). Other chemicals, if not specified, were all commercially available and used as received. The deionized water, with a resistivity of 18.2 MΩ cm^−1^, was obtained from Milli-Q Gradient System (Millipore, Bedford, MA, USA) and used for all of the experiments.

### Synthesis of cationic Pluronic F127 based nanosystem (FUP)

FUP were synthesized through self-assembly method. Briefly, 10 mg of UA, 20 mg of Pluronic F127 and 4 mg of C_18_-PEI were dissolved in 2 mL of ethyl alcohol. Then the mixture was dripped into water slowly under continuous sonication for 5 min to obtain the drug-loaded nanomicelles. The nanomicelles were filtered to remove unloaded free drug aggregates, and then washed and concentrated using an Amicon Ultra-4 centrifugal filter (Millipore) with a molecular weight cut off of 10 kDa to remove ethyl alcohol.

### Characterization

The morphology of the FUP was characterized by transmission electron microscopy (TEM, FEI Company, Hillsboro, OR) operating at an accelerating voltage of 200 kV, after stained with phosphotungstic acid. The hydrodynamic diameters and surface charge of FUP were measured by a Zetasizer Nano ZS (Malvern Instruments, Southborough, MA). Fourier transform infrared (FT-IR) spectra were obtained with a Fourier transform infrared spectrometer (Perkin-Elmer, Spectrum-2000) over the spectral region of 400 cm^−1^ to 4000 cm^−1^. The tablets for the FT-IR experiment were prepared by grinding the samples with KBr and compressing the powders into a transparent tablet.

### Analysis of UA loading and encapsulation

UA content in FUP was determined using a HPLC system (Agilent Technologies, 1260 LC) consisting of a tunable absorbance detector. For determination of UA, the mobile phase consisted of methanol/water/triethylamine (90/10/0.5, v/v/v). A Zorbax Eclipse XDB-C_18_ column was used with a flow rate of 0.6 mL min^−1^ and the detection wavelength was 210 nm. The column temperature was maintained at 25 °C. To determine drug loading content (DLC) and drug loading efficiency (DLE), the freeze-dried products were dissolved in methanol, then the supernatant was collected by centrifugation at room temperature (10 000 rpm for 5 min) for measuring its UA concentration.DLC (wt%) = (weight of loaded drug/weight of nanomicelles) × 100%DLE (%) = (weight of loaded drug/weight of feeding drug) × 100%

To investigate the drug release behaviour of UA from FUP, 0.5 mL of FUP was transferred into dialysis bag with 7.5 mL of PBS outside and incubated at 37 °C. At determined time intervals, 4 mL of PBS out of the dialysis bag was withdrawn while the same volume of counterpart PBS was added to the residual composite solutions. The amount of released UA in the supernatant was analysed by HPLC.

### Cell culture

The human colorectal cancer cell line of HCT-116 and HCT-8 and the mouse embryo fibroblast cell line of NIH3T3 obtained from Cell Bank of the Chinese Academy of Sciences (Shanghai, China) were cultured as a monolayer in RPMI-1640 medium supplemented with 10% fetal bovine serum and 1% penicillin–streptomycin at 37 °C in a humidified atmosphere (5% CO_2_ in air).

### MTT assay

The *in vitro* cytotoxicity of our nanomicelles was assessed by the standard MTT assay. Typically, HCT-116 and HCT-8 cells were seeded in a 96-well plate with a seeding density of 1 × 10^5^ cells per well for 12 hours. Then the cells were incubated with free UA or FUP with various concentrations. After 24 hours of incubation, the medium was removed, and 100 μL of MTT (0.5 mg mL^−1^ in PBS) was added for further incubation for 4 hours, the purple-blue formazan precipitate was dissolved in 100 μL of DMSO. The absorbance of the solution in each well was measured using an ELISA reader (Model ELX800, BioTek, USA) with the wavelength at 490 nm. The proliferation of cells was determined by the absorption intensity. Cell viability was calculated as follows: Cell viability (%) = OD_test_/OD_control_ × 100%, where OD_test_ and OD_control_ are the absorbance values for the treated cells and the untreated control cells, respectively. The OD_test_ and OD_control_ values were obtained after subtracting the absorbance of DMSO. All tests were performed in quadruplicate. Cell viability graphs were plotted as the UA concentration. Meanwhile, the *in vitro* cytotoxicity of blank vehicles of F127/C_18_-PEI was also evaluated on NIH3T3, HCT-116 and HCT-8 cells by using the same methods.

### Cellular uptake determination

HCT-116 and HCT-8 cells were seeded in a 6-well plate at 3 × 10^5^ cells per well, the medium was added with free UA or FUP at the equivalent drug concentration of 10 μM. After incubation for a relative short time of 4 hours to guarantee that the released drug would not induce large percentage of cell death, the cells were trypsinized and then broken by using a Branson Sonifier. The drug in cracked cells dispersion was extracted out though the addition of CH_3_OH, the mixture was centrifuged and the supernatant was collected to determine the UA concentration by using HPLC.

### Colony formation assay

For this study, HCT-116 and HCT-8 cells were seeded in a 6-well plate at 3 × 10^5^ cells per well for 12 hours, the medium was added with free UA or FUP at the equivalent drug concentration of 10 μM. After incubation for 24 hours, the cells were trypsinized and resuspended in fresh medium before reseeded in 6-well plates with a seeding density of 1000 cells per well. The cells were further incubated for 10 days with the medium replaced several times. At the end of incubation, cell colonies were fixed with 10% formaldehyde prior to be stained with 0.01% crystal violet.

### Cell cycle analysis

HCT-116 and HCT-8 cells were treated with free UA and FUP for 24 hours as indicated above. Afterwards, the cells were harvested and adjusted to a concentration of 2 × 10^5^ cells per mL. The cell cycle progression was determined through flow cytometric analysis using a Cell Cycle Assay Kit. According to the manufactures' instruction, the cells were fixed in 70% ethanol at 4 °C overnight. The fixed cells were washed twice with cold phosphate-buffered saline (PBS) and then incubated for 30 min with ribonuclease (8 μg mL^−1^) and PI (10 μg mL^−1^). The fluorescent signal was detected through the FL2 channel, and the proportion of DNA in various phases was analysed using ModFit LT version 3.0 (Verity Software House, Inc., Topsham, ME, USA).

### Cell apoptosis determination

HCT-116 and HCT-8 cells were treated with UA and FUP for 24 hours as indicated above. Annexin V/propidium iodide (PI) staining were used together with fluorescence-activated cell sorting (FACS) caliber (Becton–Dickinson) to determine the cell apoptosis. The detection was operated according to the manufacturer's instructions. After 24 hours of incubation, cells were rinsed, trypsinized, and resuspended in binding buffer, and stained with fluorescein isothiocyanate (FITC) labeled annexin V/PI. The percentage of early apoptotic (positive for annexin V), late apoptotic (double-positive for annexin V and propidium iodide) and necrotic (positive for propidium iodide) cells were analyzed by fluorescence-activated cell sorting (FACS) method.

### Western blot analysis

HCT-116 and HCT-8 cells were treated with UA and FUP for 24 hours as indicated above. Afterwards, cells were rinsed with PBS buffer for three times and lysed with RIPA lysis buffer. The total proteins were extracted from the cells and resolved electrophoretically through 15% SDS-PAGE and then transferred onto PVDF membrane. The membranes were blocked overnight at 4 °C before incubated with mouse antibody against Fas, FasL, caspase-8, caspase-3 or β-actin, while β-actin was used as an internal standard to normalize protein expression. After incubated with HRP-labeled goat anti-mouse antibodies at 37 °C for 1 h, the proteins were analyzed by using BeyoECL Plus. Image Lab™ Software (Version 3.0) was used for densitometric analysis and quantification of western blots.

### Statistical analysis

Statistical analysis was performed *via* the SPSS package for Windows (version 17.0, SPSS Inc., Chicago, IL, USA) by using Fisher's least significant difference (LSD) test or one-way ANOVA. *p < 0.05 was considered as significance in the present study.

## Results and discussion

### Synthesis and characterization of FUP

**Fig. 1:**
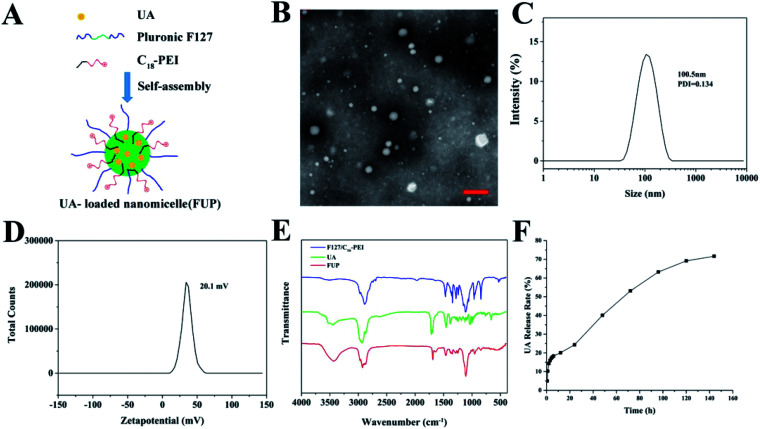
(A) Schematic view of the formation of FUP. (B) TEM image of the FUP, scale bar is 200 nm. Size distribution (C) and zeta potential (D) of the FUP, as determined by the DLS analysis. (E) The FT-IR spectra of free UA (green curve), FUP (red curve) and blank vehicles of F127/C_18_-PEI. (F) *In vitro* release of UA from FUP.

The synthesis procedure of FUP is schematically illustrated in Fig. 1A. FUP was synthesized through self-assembly of F127, UA and C_18_-PEI, and the obtained nanomicelles were characterized by TEM and DLS. As shown in Fig. 1B, FUP showed a spherical morphology with a size in the range of 30–150 nm. DLS studies (Fig. 1C) revealed that the average hydrodynamic size of micelles was around 100 nm, which was consistent with the TEM results. The zeta potential (Fig. 1D) of the nanomicelles was measured to be 20.1 mV, which was ascribed to the positive charged groups (–NH_2_ and –NH– in C_18_-PEI) on their surface. Fourier transform infrared spectroscopy (FT-IR) was employed to further confirm the incorporating of UA into the nanomicelles. As shown in Fig. 1E, compared with blank vehicles of F127/C_18_-PEI, a new peak at 1690 cm^−1^ and 3429 cm^−1^ assigned to the C=O groups and C–OH of UA was observed in the spectra of FUP, indicating that UA was successfully integrated into FUP.

To investigate the stability of the nanoparticles, the FUP were stored in PBS for 8 days to observe the hydrodynamic size change. As shown in Fig. S1,† there were no apparent aggregation or precipitation even storied for 8 days during our observation, with an average hydrodynamic size maintained below 150 nm, implying well colloid stability of our nanoparticles in physiological conditions.

### UA-loaded efficiency an *in vitro* UA release

To quantitatively investigate the UA loading amount in the FUP, the standard curve of the absorbance measured by HPLC of UA at 210 nm *versus* the UA concentration was plotted with a very nice linear correlation (with the correlation coefficient of 0.999). Based on this standard curve, the DLC and DLE were respectively calculated to be 36.64% and 15.2% according to the protocol in “methods” section, which is more than the other nano-carrier such as mPEG-PCL (4.75 ± 0.45%) and PLGA (8 ± 1.23%).^23,24^ The result of the UA *in vitro* release from FUP was shown in Fig. 1F. A burst release was observed at the early stage (within 8 hours) with more than 20% of drug release due to the large concentration gradient between FUP dispersion in dialysis bag and outside PBS. Afterwards, the release rate became slower, and reached a platform with a value of 71.7%, after 144 h. This means that a part of the incorporated drug is kept stable within the vehicles, and thus FUP can release their payload drug though a slow manner.

### *In vitro* cytotoxicity of FUP against colorectal cancer cells

Nontoxicity or low toxicity is a vital index of any nanomaterial applied in biomedicine. The *in vitro* cytotoxicity of F127/C_18_-PEI is assessed on NIH3T3 cells by using MTT assay. The cells are incubated with our nanocomposites at a series of gradient concentrations for 24 or 48 hours, and the cell viability results are shown in Fig. S2.† Blank vehicles of F127/C_18_-PEI showed a very low cytotoxic effect on the cells; even at a high dose of 50 μg mL^−1^, the cells remain more than 85% viable after 48 h incubation.

### *In vitro* cytotoxicity of FUP against colorectal cancer cells

**Fig. 2:**
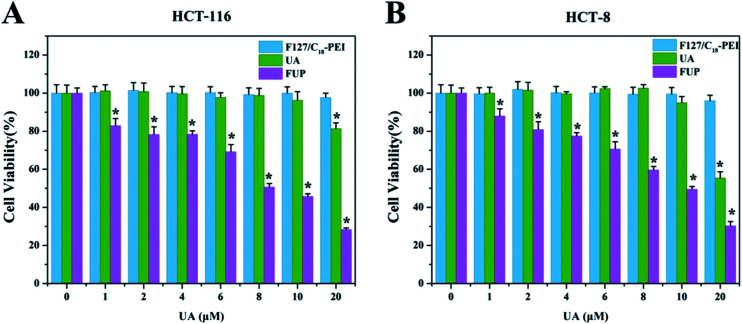
*In vitro* cytotoxicity of blank vehicle of F127/C_18_-PEI, free UA and FUP against HCT-116 cells (A) and HCT-8 cells (B), after 48 hours incubation. Data are expressed as the mean ± standard deviation (error bars) from at least three independent experiments. *p* < 0.05.

The inhibitory effect of F127/C_18_-PEI, free UA and FUP on the CRC cells were determined by MTT assay. As shown in Fig. 2, after 48 h incubation, FUP exhibited an obvious dose-dependent cytotoxicity in both HCT-116 and HCT-8 cells, and less than 30% of the cells remained alive at the UA concentration of 20 μM, which is much lower than that cells treated free FUP at the equivalent dose. This superiority might be resulted from the enhanced uptake of the cationic FUP and the sustained release of UA in the cancer cells.^25^ The IC_50_ of FUP in HCT-116 and HCT-8 was 9.26 μM and 10.15 μM, respectively. It is noteworthy that blank vehicles of F127/C_18_-PEI of 0–16 μg mL^−1^ with equivalent UA concentrations of 0–20 μM had negligible inhibition effect on cell proliferation, confirming the safe nature of our nanovehicles. Indeed, at high dosage ranged from 40–160 μg mL^−1^, F127/C_18_-PEI with slightly positive charge showed significant cytotoxicity (Fig. S3†). However, the dosage of F127/C_18_-PEI (0–16 μg mL^−1^) with equivalent UA concentrations of 0–20 μM is too low to efficiently inhibit cell proliferation. The lower cytotoxicity of our nanoparticles with slightly positive charge might be ascribed to the highly biocompatibility of F127 as a main component for encapsulating UA.^17,18^ Based on these results, the FUP concentration of 10 μM was selected to further investigated the inhibiting effect and relevant mechanisms of FUP in the following experiments.

### Cellular uptake determination

To demonstrate that the positive charge of FUP will benefit to attach to the negatively charged cell membranes for enhanced cellular uptake, here the content of UA in HCT-116 and HCT-8 cells pre-treated with FUP or non-ion micelles assembled from UA and F127 (named as FUN), was determined by using HPLC. As shown in Fig. S4,† the drug content in HCT-116 or HCT-8 cells pre-treated with FUP is much higher than that pre-treated with FUN at the same containing UA concentrations.

### Growth inhibition effect of FUP

**Fig. 3:**
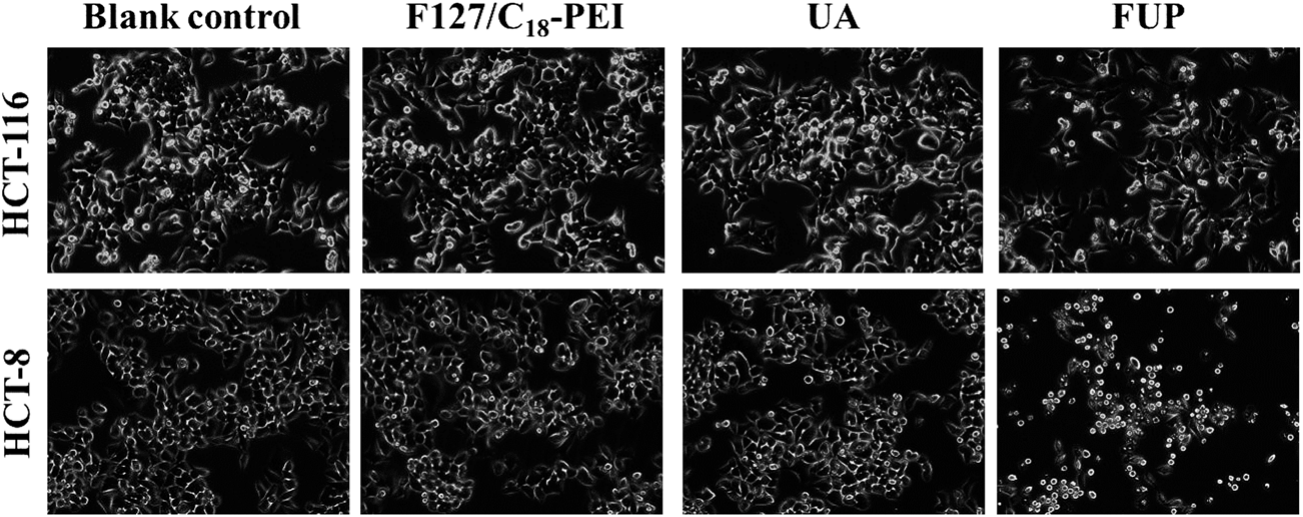
Morphological changes of HCT-116 and HCT-8 observed by phase-contrast microscopy. Photographs were taken at a magnification of 200×. Images are representative of three independent experiments.

To intuitively observe the effects of FUP on cell morphology, the appearance of the treated cells was imaged by phase contrast microscopy. Untreated or blank vehicles treated cells exhibited as a crowded and disorganized monolayer after 24 h. However, the cell density was reduced dramatically when the cells are treated with FUP, and the residual cells showed a bright shrinkage morphology (Fig. 3). Compared with the FUP group, the free UA treated cells had much less impact on the cell density and morphology.

**Fig. 4:**
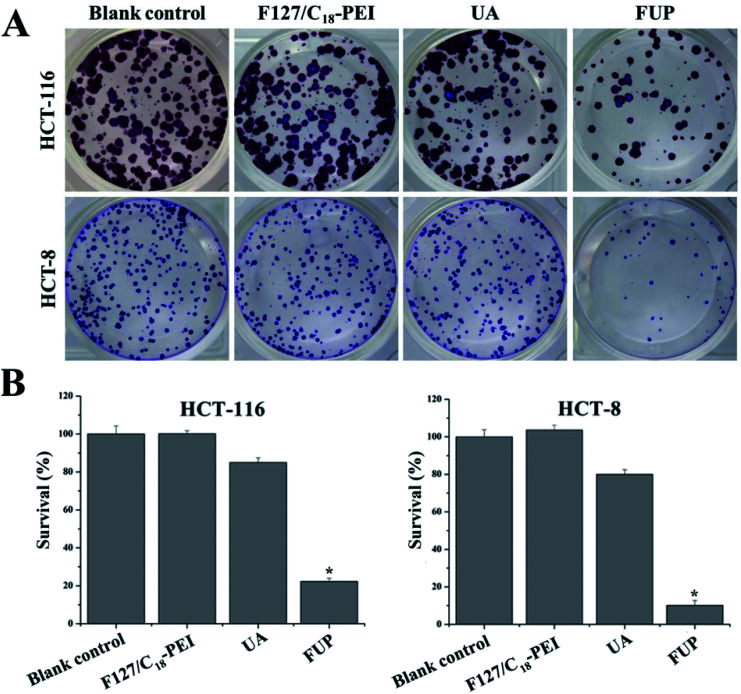
Cell survival was determined by colony formation analysis. Images are representative of three independent experiments. Bar graph representing the statistical results of colony number. Data are expressed as the mean ± standard deviation (error bars) from at least three independent experiments. *p < 0.05.

Furthermore, the cytotoxicity of FUP against HCT-116 and HCT-8 cells were also examined from the aspect of the colony formation assay. As shown in Fig. 4A, the brown or blue granules of HCT-116 and HCT-8 clones reduced dramatically after FUP treatment, and there were almost no clones observed after incubation for 24 h. Meanwhile, the number of adhered colonies in each dish was counted and then normalized with that blank control group to calculate the survival rate. As shown in Fig. 4B, after FUP treatment, the survival rate in HCT-116 and HCT-8 cells was less than 20%, while the adhered colony ratio was above 80% in free FUP group. This result is consistent with the MTT assay which demonstrate our FUP has better cell inhibition effect than free UA.

### Effect of FUP on cell cycle

**Fig. 5:**
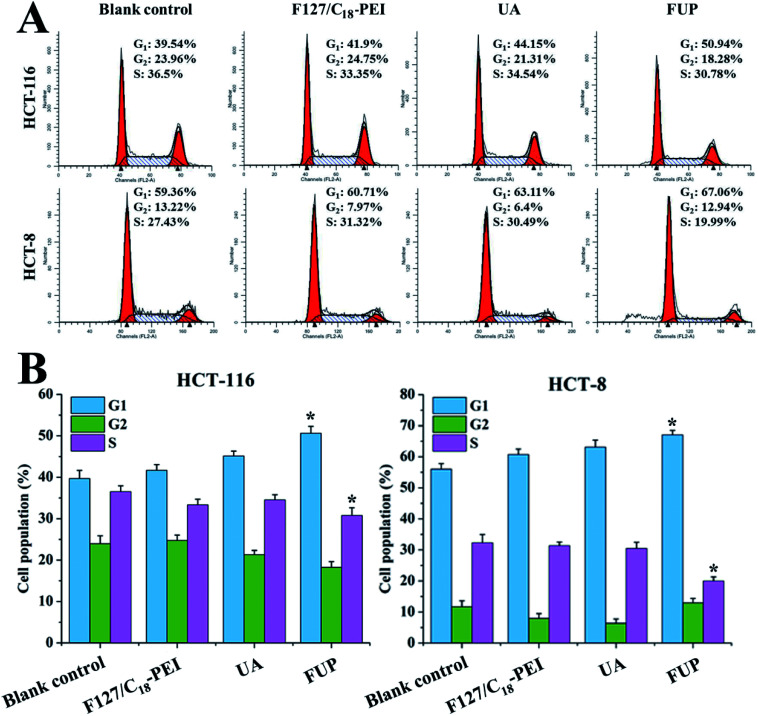
(A) Representative flow cytometry histograms of cell cycle analysis of HCT-116 and HCT-8 cells after incubation with F127/C_18_-PEI, UA and FUP. (B) The percentage of cells in G1, S and G2 calculated using Multicycle software. Data are expressed as the mean ± standard deviation (error bars) from at least three independent experiments. *p < 0.05.

To determine whether the cell growth inhibition of FUP is mediated by interfering with cell cycle progression, HCT-116 or HCT-8 after FUP treatment was stained with propidium iodide and analyzed by flow cytometer. As shown in Fig. 5, FUP treatment resulted in significant G1-phase cell cycle arrest, compared with blank vehicle of F127/C_18_-PEI and free UA. 10 μM FUP increased the population of G1 phase from 39.7 ± 2.0% to 50.6 ± 4.7%, while free UA treated cell only increased to 45.1 ± 1.2%. The results revealed that the FUP showed a cell cycle arrest at the G1 checkpoint.

### Effect of FUP on cell apoptosis

**Fig. 6:**
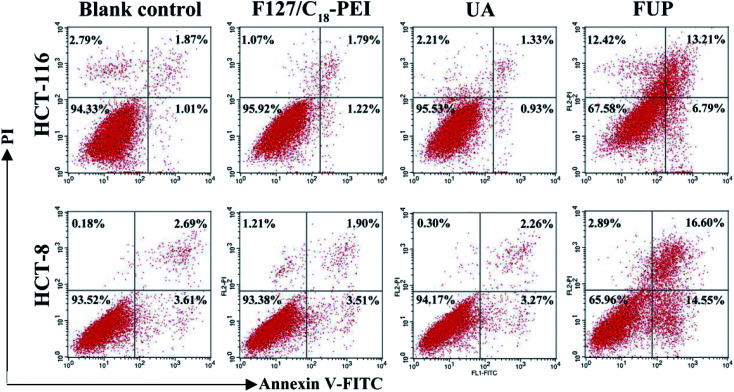
The effect of FUP on apoptosis in HCT-116 and HCT-8 cells after incubation with F127/C_18_-PEI, UA and FUP. The cells apoptosis were determined by FACS analysis by using annexin V-FITC and PI staining.

In order to study the mechanism of the growth suppressive activity of FUP, its effect on apoptosis in HCT-116 and HCT-8 cells was assessed *via* annexin V-FITC/PI staining incorporated with FACS analysis. The stained cells are divided into four subgroups, lower left quadrant represent viable cells which are negatively stained for both annexin V-FITC and PI, lower right quadrant represent early apoptosis event where the cells stained by annexin V-FITC alone, upper right quadrant is the late apoptosis event with cells stained with both annexin V-FITC and PI, and upper left quadrant is the region of necrotic event with cells only stained by PI.^26,27^ As shown in Fig. 6, the majorities of HCT-116 and HCT-8 cells were both localized in the lower left quadrant with more than 90% of the viable cells in the control group, indicating no apparent cell death. Compared with control group, the viable cells were decreased dramatically after incubation with FUP. At contained UP concentration of 10 μM, the percentages of survive cells in FUP group decreased to be 67.58% (HCT-116) and 65.96% (HCT-8), accompanied with the apoptosis ratio increased to 20% (HCT-116) and 31.15% (HCT-8), respectively. However, blank vehicle of F127/C_18_-PEI and free UA has little influence on cell apoptosis. This result is consistent with the MTT assay and indicates FUP can effectively block cancer cell proliferation *via* inducing cell apoptosis.

### Expression of Fas, FasL cleaved caspase-8 and cleaved caspase-3 in HCT-116 and HCT-8 cells regulated by FUP

**Fig. 7:**
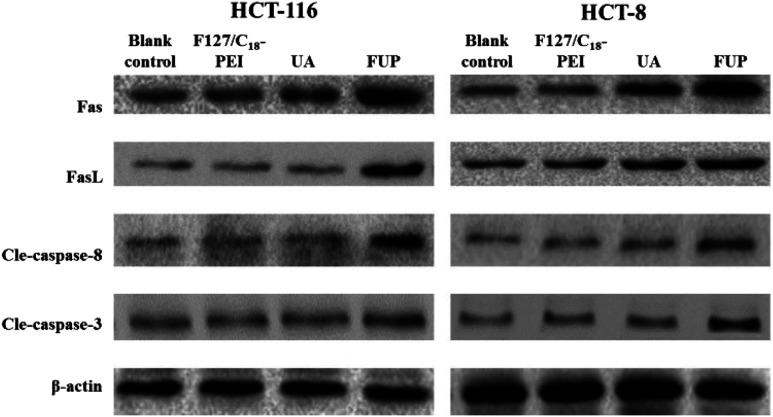
The levels of Fas, FasL cleaved caspase-8 and cleaved caspase-3 in HCT-116 and HCT-8 cells, determined by western blotting. Images are representative of three independent experiments.

To further explore the apoptosis pathway of cancer cell after FUP treatment, we performed western blot analysis to determine the expression of cleaved caspase-8, cleaved caspase-3, Fas and FasL in protein levels, respectively. The reason of choosing this protein is: extracellular protein FasL is the ligand to the Fas, which is a trans-membrane protein, belongs to the tumor necrosis factor receptor superfamily member, the Fas–FasL interaction plays an important role in activation of the death receptor apoptotic pathway. Their binding induce caspase-8 cleaved (activation of caspase-8), subsequently caspase-3 cleaved (activation caspase-3). It has reported that UA could induce cell apoptosis by regulating Fas, FasL, caspase-8 and caspase-3.^27,28^ Accordingly, the protein expression patterns showed that FUP treatment increasing protein levels of Fas, FasL cleaved caspase-8 and cleaved caspase-3, while no significant changes were found in F127/C_18_-PEI and UA groups (Fig. 7).

## Conclusions

In this work, we prepared a cationic nanomicelle derived from Pluronic F127 as a delivery vehicle of ursolic acid for enhanced colorectal cancer treatment. The size of FUP was 30–150 nm and the surface charge of FUP exhibited a positive zeta potential about 20.1 mV. Cellular uptake of nanomicelles by colorectal cancer cells was enhanced due to the positive charge of surface. The inhibition effects of FUP on cell viability, colony formation, proliferation and inducing cell apoptosis were better than free UA, and the antitumor mechanism might be related with the regulation of Fas/FasL and activation of caspase-8 and caspase-3. Hence, in view of the advantages mentioned above, the prepared FUP could serve as a promising therapeutic agent for colorectal cancer therapy.

## Conflicts of interest

There are no conflicts to declare.

## Supplementary Material

RA-008-C8RA01071D-s001

## References

[cit1] Torre L. A., Bray F., Siegel R. L., Ferlay J., Lortet-Tieulent J., Jemal A. (2012). Journal for Clinicians.

[cit2] Siegel R. L., Miller K. D., Fedewa S. A., Ahnen D. J., Meester R. G. S., Barzi A., Jemal A. (2017). Ca-Cancer J. Clin..

[cit3] Gao F. H., Hu X. H., Li W., Liu H., Zhang Y. J., Guo Z. Y., Xu M. H., Wang S. T., Jiang B., Liu F., Zhao Y. Z., Fang Y., Chen F. Y., Wu Y. L. (2010). BMC Cancer.

[cit4] Saltz L. B., Clarke S., Díaz-Rubio E., Scheithauer W., Figer A., Wong R., Koski S., Lichinitser M., Yang T. S., Rivera F., Couture F., Sirzén F., Cassidy J. (2008). J. Clin. Oncol..

[cit5] van Erning F. N., Janssen-Heijnen M. L. G., Wegdam J. A., Slooter G. D., Wijsman J. H., Vreugenhil A., Beijers T. A. J. M., van de Poll-Franse L. V., Lemmens V. E. P. P. (2017). Clin. Colorectal Cancer.

[cit6] Kokotis P., Schmelz M., Kostouros E., Karandreas N., Dimopoulos M. A. (2016). Clin. Colorectal Cancer.

[cit7] Chau I., Cunningham D. (2002). Br. Med. Bull..

[cit8] Conroy T., Paillot B., François E., Bugat R., Jacob J. H., Stein U., Nasca S., Metges J. P., Rixe O., Michel P., Magherini E., Hua A., Deplanque G. (2005). J. Clin. Oncol..

[cit9] Normile D. (2003). Science.

[cit10] Xu W., Towers A. D., Li P., Collet J. P. (2006). Eur. J. Cancer.

[cit11] Baricevic D., Sosa S., Della Loggia R., Tubaro A., Simonovska B., Krasna A., Zupancic A. (2001). J. Ethnopharmacol..

[cit12] Li J., Guo W. J., Yang Q. Y. (2002). World J. Gastroenterol..

[cit13] Chen H., Gao Y., Wang A., Zhou X., Zheng Y., Zhou J. (2015). Eur. J. Med. Chem..

[cit14] Reyes-Zurita F. J., Rufino-Palomares E. E., Lupiáñez J. A., Cascante M. (2009). Cancer Lett..

[cit15] Zhang H., Zheng D., Ding J., Xu H., Li X., Sun W. (2015). Int. J. Nanomed..

[cit16] Cabral H., Kataoka K. (2014). J. Controlled Release.

[cit17] Kataoka K., Harada A., Nagasaki Y. (2001). Adv. Drug Delivery Rev..

[cit18] Maeda H., Nakamura H., Fang J. (2013). Adv. Drug Delivery Rev..

[cit19] Pelaz B., del Pino P., Maffre P., Hartmann R., Gallego M., Rivera-Fernández S., de la Fuente J. M., Nienhaus G. U., Parak W. J. (2015). ACS Nano.

[cit20] Hama S., Itakura S., Nakai M., Nakayama K., Morimoto S., Suzuki S., Kogure K. (2015). J. Controlled Release.

[cit21] Tang S., Meng Q., Sun H., Su J., Yin Q., Zhang Z., Yu H., Chen L., Gu W., Li Y. (2017). Biomaterials.

[cit22] Wu L., Wu M., Lin X., Zhang X., Liu X., Liu J. (2017). J. Mater. Chem. B.

[cit23] Zhang H., Li X., Ding J., Xu H., Dai X., Hou Z., Zhang K., Sun K., Sun W. (2013). Int. J. Pharm..

[cit24] Baishya R., Nayak D. K., Kumar D., Sinha S., Gupta A., Ganguly S., Debnath M. C. (2016). Pharm. Res..

[cit25] Le Garrec D., Gori S., Luo L., Lessard D., Smith D. C., Yessine M. A., Ranger M., Leroux J. C. (2004). J. Controlled Release.

[cit26] Zhang D., Zheng A., Li J., Wu M., Cai Z., Wu L., Wei Z., Yang H., Liu X., Liu J. (2017). Adv. Sci..

[cit27] Zhang D., Zheng A., Li J., Wu M., Wu L., Wei Z., Liao N., Zhang X., Cai Z., Yang H., Liu G., Liu X., Liu J. (2017). Theranostics.

[cit28] Zhang L., Cai Q., Lin J., Fang Y., Zhan Y., Shen A., Wei L., Wang L., Peng J. (2014). Mol. Med. Rep..

