# Supplementary material for: Cationic nanomicelles derived from Pluronic F127 as delivery vehicles of Chinese herbal medicine active components of ursolic acid for colorectal cancer treatment
Source: RSC Adv. 2018 Apr 27;8(29):15906–14. doi: 10.1039/c8ra01071d (PMC9080071; doi:10.1039/c8ra01071d)
Supplement: RA-008-C8RA01071D-s001 [file RA-008-C8RA01071D-s001.pdf]

Electronic Supplementary Material (ESI) for RSC Advances.
This journal is © The Royal Society of Chemistry 2018

Eelectronic Supplementary Information (ESI)

Cationic nanomicelles derived from Pluronic F127 as delivery vehicles of Chinese herbal medicine active components of ursolic acid for colorectal cancer treatment

Zhaokun Yan,[‡a] Qingtang Wang,[‡b,c] Xiaolong Liu,[b,c] Jun Peng,[a,d] Qin Li,[b,c] Ming Wu,[*b,c] Jiumao Lin[*a,d]

[a] Academy of Integrative Medicine, Fujian University of Traditional Chinese Medicine, Fuzhou 350122, P.R. China. E-mail: jiumaolin@hotmail.com

[b] The United Innovation of Mengchao Hepatobiliary Technology Key Laboratory of Fujian Province, Mengchao Hepatobiliary Hospital of Fujian Medical University, Fuzhou 350025, P. R. China. E-mail: wmmj0419@163.com

[c] The Liver Center of Fujian Province, Fujian Medical University, Fuzhou 350025, P. R. China.

[d] Fujian Key Laboratory of Integrative Medicine on Geriatrics, Fujian University of Traditional Chinese Medicine, Fuzhou, Fujian 350122, P.R. China

‡ These authors contribute equally to this work.

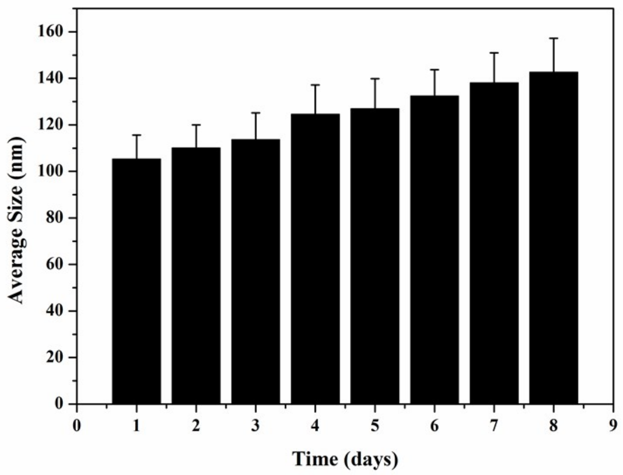

Figure S1. Hydrodynamic size of FUP in PBS measured by DLS, after storied for different times ranged from 1 d to 8 d.

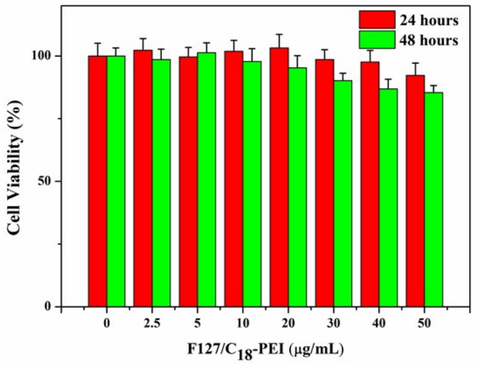

Figure S2. *In vitro* cytotoxicity of blank vehicle of F127/C_{18}-PEI against NIH3T3 cells, after 24 or 48 hours of incubation. Data are expressed as the mean ± standard deviation (error bars) from at least three independent experiments.

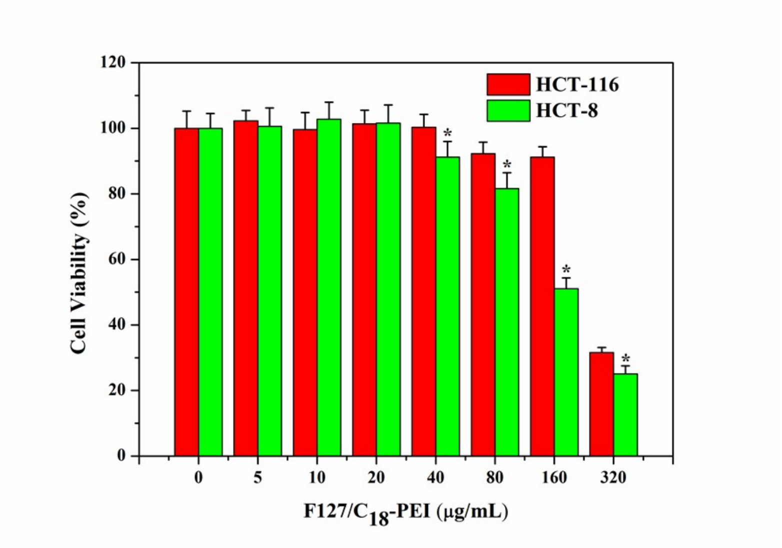

Figure S3. *In vitro* cytotoxicity of cationic blank vehicle of F127/C_{18}-PEI against HCT-116 or HCT-8 cells, after 48 hours of incubation. Data are expressed as the mean ± standard deviation (error bars) from at least three independent experiments.

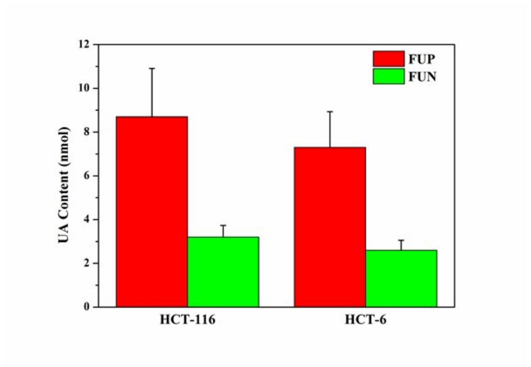

Figure S4. UA content in HCT-116 and HCT-8 cells incubated with FUP or FUN for 4 hours.
